# Constructing ferroptosis-related competing endogenous RNA networks and exploring potential biomarkers correlated with immune infiltration cells in asthma using combinative bioinformatics strategy

**DOI:** 10.1186/s12864-023-09400-7

**Published:** 2023-05-31

**Authors:** Shao-Tian Chen, Nan Yang

**Affiliations:** Department of Pediatrics, Shengjing Hospital of China Medical University, 36 Sanhao Street Liaoning Province, 110004 Shenyang, China

**Keywords:** Asthma, Diagnostic biomarker, CeRNA network, Ferroptosis, Immune infiltration analysis

## Abstract

**Background:**

Asthma is a common chronic respiratory disease worldwide. Recent studies have revealed the critical effects of the ceRNA network and ferroptosis on patients with asthma. Thus, this study aimed to explore the potential ferroptosis-related ceRNA network, investigate the immune cell infiltration level in asthma through integrated analysis of public asthma microarray datasets, and find suitable diagnostic biomarkers for asthma.

**Methods:**

First, three asthma-related datasets which were downloaded from the Gene Expression Omnibus (GEO) database were integrated into one pooled dataset after correcting for batch effects. Next, we screened differentially expressed lncRNAs (DElncRNAs) between patients and healthy subjects, constructed a ceRNA network using the StarBase database and screened ferroptosis–related genes from the predicted target mRNAs for Disease Ontology (DO), Gene Ontology (GO), and Kyoto Encyclopedia of Genes and Genomes (KEGG) enrichment analyses. We also performed Gene Set Enrichment Analysis (GSEA) and Gene Set Variation Analysis (GSVA) on the batch effect-corrected mRNA expression profile. Then, Least Absolute Shrinkage and Selection Operator (LASSO) regression was used to screen potential diagnostic biomarkers, and the diagnostic efficacy was assessed using a receiver operating characteristic (ROC) curve. Finally, we determined the proportion of 22 immune cells in patients with asthma using CIBERSORT and investigated the correlation between key RNAs and immune cells.

**Results:**

We obtained 19 DElncRNAs, of which only LUCAT1 and MIR222HG had corresponding target miRNAs. The differentially expressed ferroptosis-related genes were involved in multiple programmed cell death-related pathways. We also found that the mRNA expression profile was primarily enriched in innate immune system responses. We screened seven candidate diagnostic biomarkers for asthma using LASSO regression (namely, BCL10, CD300E, IER2, MMP13, OAF, TBC1D3, and TMEM151A), among which the area under the curve (AUC) value for CD300E and IER2 were 0.722 and 0.856, respectively. Finally, we revealed the infiltration ratio of different immune cells in asthma and found a correlation between LUCAT1, MIR222HG, CD300E, and IER2 with some immune cells.

**Conclusion:**

This study explored a potential lncRNA-miRNA-mRNA regulatory network and its underlying diagnostic biomarkers (CD300E and IER2) in asthma and identified the immune cells most associated with them, providing possible diagnostic markers and immunotherapeutic targets for asthma.

**Supplementary Information:**

The online version contains supplementary material available at 10.1186/s12864-023-09400-7.

## Introduction

Asthma is a chronic airway inflammatory disease, and the Global Asthma Network 1 (GAN1) study reported that the prevalence rates of asthma in children and adults were 10.5% and 4.4%, respectively [1, 2]. The overall global burden of asthma is still substantial. The main characteristics of asthma include airway inflammation, hyperresponsiveness, mucus hypersecretion, and airway remodeling [3]. However, the pathogenesis of asthma involves complex gene-environment interactions. Furthermore, current treatment options for asthma are primarily directed at symptom control rather than altering the prognostic endpoints of the disease, with a subset of patients with asthma whose symptoms cannot be controlled [4]. Therefore, identifying potential asthma biomarkers utilizing bioinformatic analysis might provide insights into the pathogenesis of asthma and determine new therapeutic targets.

The effect of competing endogenous RNAs (ceRNAs) on asthma has attracted the interest of researchers over recent years. Hence, further studies on ceRNAs might help to find new mechanisms for asthma treatment. CeRNAs, as a newly discovered mechanism of gene expression regulation, have an elaborate and complex regulatory network involving several RNA molecules, such as long non-coding RNAs (lncRNAs), pseudogenes, circular RNAs (circRNAs), viral RNAs, and mRNAs [5]. The present study aims to construct a lncRNA–miRNA–mRNA regulatory network. LncRNAs and mRNAs have miRNA response elements (MREs). LncRNAs indirectly regulate mRNA expression levels and cellular functions by competitively binding MREs. Furthermore, the trend in expression levels is consistent across ceRNAs [6]. A growing number of studies have revealed that lncRNAs could regulate different cellular processes (for example, inflammation [7], proliferation [8], apoptosis [9], migration, and epithelial–mesenchymal transition) through the lncRNA-miRNA-mRNA axis, thereby regulating asthma progression. Therefore, our study focuses on the lncRNA–miRNA–mRNA axis in asthma through bioinformatic analysis to investigate the molecular regulatory mechanism involved in peripheral blood mononuclear cells (PBMCs) among patients with asthma.

Ferroptosis has garnered interest of researchers over the past few years, and research on its role in the pathogenesis of asthma has gained momentum. The ferroptosis pathway, first proposed in 2012, is an iron-dependent novel form of programmed cell death, which varies from apoptosis, cell necrosis, and cell autophagy [10]. Ferroptosis could be triggered by iron-catalyzed lipid peroxidation, which is mediated by non-enzymatic (Fenton reaction) and enzymatic (lipoxygenases [LOXs]) mechanisms [11]. The excessive release of oxidized lipid mediators increases the activity of cyclooxygenase (COX) and LOX, accelerates the metabolism of arachidonic acid, promotes the secretion of inflammatory signaling molecules [12], and mediates inflammatory responses and immune cell chemotaxis. Studies have shown that the phosphatidylethanolamine-binding protein 1/15 LOX (PEBP1/15-LO) complex is a critical regulator of ferroptosis capable of stimulating IL-13/ IL-4-induced Th-cell inflammation. The colocalization level of PEBP1 and 15-LO in human airway epithelial cells (HAECs) of patients with asthma is higher than that in those of the normal population, indicating the likelihood of ferroptosis in HAECs of patients with asthma [13, 14]. Moreover, increased lipid peroxidation and ROS production levels as well as significantly reduced glutathione peroxidase 4 (GPX4) and solute carrier family 7 member 11 (SLC7A11) protein levels have been reported in lung tissues of a murine house dust mite–induced asthma model,, indicating an increase in ferroptosis in lung tissues of asthmatic mice [15]. Therefore, treatment of asthmatic mice with ferroptosis inhibitors such as deferoxamine (DFO) and ferrostatin-1 (Fer-1) significantly reduces airway inflammation [16], suggesting that ferroptosis plays a crucial role in airway epithelial cells of animal models with asthma.

Studies have revealed that Th1/Th2-mediated immune imbalance serves as the predominant mechanism of airway inflammatory response in asthma [17] and that various immune cells are involved in the pathogenesis of asthma, including eosinophils, mast cells, dendritic cells (DCs), macrophages, neutrophils, T lymphocytes, B lymphocytes, and innate lymphocytes [18]. Moreover, information on the ratio of different infiltrated immune cells can promote the classification and diagnosis of patients with asthma [19, 20]. For instance, asthma is classified as eosinophilic asthma, neutrophilic asthma, mixed granulocytic asthma, and paucigranulocytic asthma based on the analysis of inflammatory cell count in induced sputum. However, only few studies have explored whether other immune cells have diagnostic value. Therefore, identifying the ratios of different immune cells in asthma could help us understand asthma progression and establish an efficient diagnosis and personalized treatment strategy.

**Fig. 1:**
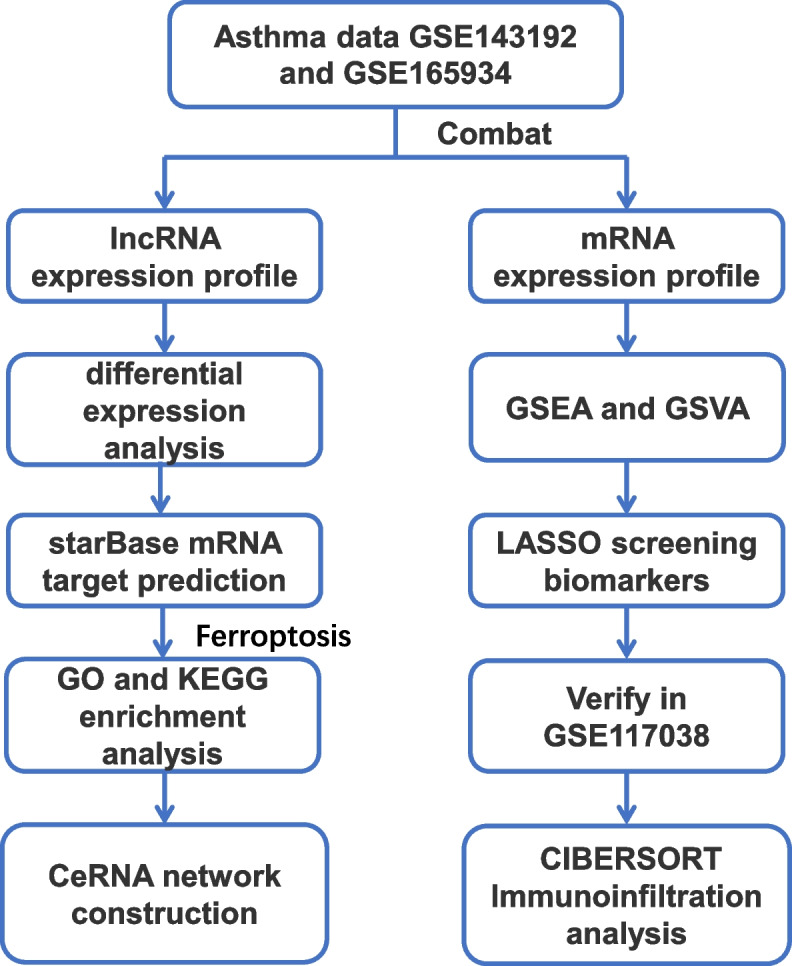
Complete flow chart of our research

In this study, we obtained three microarray expression datasets of PBMC from patients with asthma and healthy individuals by screening the Gene Expression Omnibus (GEO) database, including two training sets (GSE143192/GSE165934) and one validation set (GSE117038). After removing the batch effect (n_asthma_ = 14, n_normal_ = 13), the training sets were combined to obtain lncRNA and mRNA expression profiles. After screening the differentially expressed lncRNAs against the lncRNA expression profile, target mRNAs were predicted using the StarBase database and intersected with ferroptosis-related genes for Disease Ontology (DO), Gene Ontology (GO), and Kyoto Encyclopedia of Genes and Genomes (KEGG) enrichment analyses to construct the ceRNA network. Then, we performed Gene Set Enrichment Analysis (GSEA) and Gene Set Variation Analysis (GSVA) analyses against the mRNA expression profile and screened key genes using the Least Absolute Shrinkage and Selection Operator (LASSO) machine learning algorithm. Afterward, receiver operating characteristic (ROC) curve analysis was applied to screen genes (as diagnostic markers) with high area under the curve (AUC) values. Finally, CIBERSORT was applied to perform immune infiltration analysis, while key lncRNAs and mRNAs were correlated with different immune cell infiltration levels. The experimental flow chart of this study is shown in Fig. 1, and the details of all data sets are shown in Table 1.

**Table 1:** Summary table of GEO dataset information

Data set	Normal	Asthma	Total
GSE143192	4	4	8
GSE165934	9	10	19
GSE117038	3	9	12

## Materials and methods

### Data downloading and processing

We downloaded three public asthma-related peripheral blood mononuclear cell gene expression dataset profiles from the Gene Expression Omnibus database managed by the National Center for Biotechnology Information [21] (https://www.ncbi.nlm.nih.gov). The profiles included training sets GSE143192 [7] (n_asthma_ = 4, n_normal_ = 4) and GSE165934 (n_asthma_ = 10, n_normal_ = 9) and validation set GSE117038 [22] (n_asthma_ = 9, n_normal_ = 3). The species source of these three datasets is human (Homo sapiens) with platform numbers GPL22120, GPL23126, and GPL16791. Next, we downloaded human phenotype ontology (c5.hpo.v7.2.symbols.gmt), C2 (Curated gene sets, c2.all.v7.2.symbols.gmt), and C5 (c5.go.v7.2.symbols.gmt) gene sets from the MSigDB database [23] (https://www.gsea-msigdb.org/gsea/msigdb). Finally, we compiled 453 ferroptosis–related genes (Table S1) from the GeneCards database [24] (http://www.genecards.org), FerrDb database [25] (http://www.zhounan.org/ferrdb/), and ferroptosis–related literature in the journal CELL [26].

The datasets used in this study were derived from different study types and experimental conditions, generating the batch effect. Therefore, we first extracted common lncRNAs and mRNAs from GSE143192 and GSE165934, performed batch effect correction using the Combat function in the R package “sva” [27], and finally performed multiple analyses (as described below) on the obtained integrated lncRNA and mRNA expression profile (n_asthma_ = 14, n_normal_ = 13). In addition, we evaluated data quality before and after the batch effect correction and then constructed box and Principal Components Analysis (PCA) plots of the lncRNA expression profile.

### Differentially expressed lncRNA screening and ceRNA network construction

Differential analysis of the batch effect–corrected lncRNA expression profile was performed using the R package “limma” [28], using patients with asthma and healthy individuals as the grouping criterion, a difference threshold of Log2|FC|> 1, and p-value of < 0.05. Then, volcano plots and heat maps of differentially expressed lncRNAs were generated using the R package “ggplot2” [29] and “pheatmap” respectively.

Next, we constructed a ceRNA network using the obtained differentially expressed lncRNAs with StarBase [30] (http://StarBase.sysu.edu.cn/). Most lncRNAs showed no corresponding target miRNAs, with only lung cancer-associated transcript 1 (LUCAT1) and MIR222HG being identified as predictable target genes. Therefore, we constructed ceRNA networks using only these two lncRNAs. Furthermore, the ceRNA networks were visualized using Cytoscape [31].

### Ferroptosis-related DEG enrichment analysis

The predicted target mRNAs from the ceRNA network constructed using differentially expressed lncRNAs were intersected with ferroptosis-related genes. Venn diagrams were plotted to obtain differentially expressed genes related to ferroptosis. These genes were subjected to DO [32], GO [33], and KEGG [34] enrichment analyses using the R package “clusterProfiler” [35].

### GSEA and GSVA analyses of the mRNA expression profile

The mRNA expression profile corrected for batch effect was analyzed by GSEA [36] and GSVA [37]. GSEA uses genes in a predefined gene set to assess their distribution trend based on their correlation with the phenotype, thereby determining their contribution to this phenotype. For this analysis, “c2.all.v7.2.symbols.gmt” was selected as the reference gene set. GSVA is a non-parametric unsupervised analysis primarily used to assess the enrichment profile of gene sets in microarrays and transcriptomes. This analysis evaluates whether different metabolic pathways are enriched among samples by converting the expression matrix of genes into the expression matrix of gene sets. For GSVA, Hallmarker, KEGG, and GO-related gene sets were selected as reference gene sets. Then, the GSVA score of each gene set was quantified. Finally, we used the R package “limma” to perform a variance analysis based on the grouping information of patients with asthma and normal subjects and plotted histograms of upregulated and downregulated differentially expressed gene sets sorted by t-value.

### Biomarker screening by LASSO regression

The integrated training set corrected for batch effect was first screened for key genes in asthma using the LASSO algorithm. We applied these candidate genes to the GSE117038 validation set to predict the asthma status in this cohort and validate the diagnostic validity of these candidate biomarkers. Then, ROC curves were used. The AUC values were calculated, and genes with strong ROC results (AUC value of > 0.7) were selected as diagnostic markers for asthma.

### Immune infiltration analysis by CIBERSORT and the correlation between key genes and infiltrating immune cells

CIBERSORT [38] was used to analyze the proportion of various immune cells in PBMC in patients with asthma and healthy subjects based on the principle of linear support vector regression. The Wilcoxon Rank sum-test was used in the immune infiltration analysis by CIBERSORT, and p-value < 0.05 demonstrated that the immune cell infiltration matrix was acquired. Then, we investigated the association between the key genes screened in the previous analysis and the ratio of immune cell infiltration and calculated Pearson correlation coefficients along with *p*-values (*p*-value < 0.05 as the significance threshold). Finally, we plotted lollipop plots showing the correlation among each lncRNA, mRNA, and different immune cells using the R package “ggplot2”.

### Statistical analysis

All the experiments were performed on R Software (https://www.r-project.org/ version 4.1.1). The correlation between genes and immune cells was measured by Spearman coefficient and corrected by Benjamini-Hochberg (BH) multiple test, FDR correction was performed on multiple tests to reduce false-positive rates. Due to the data species and characteristics, differences were analyzed by the Wilcoxon rank-sum test and Student’s t test for the categorical variables and the continuous variables, respectively. The two-sided test was considered statistically significant at p-value < 0.05.

## Results

### Batch effect correction of the data

**Fig. 2:**
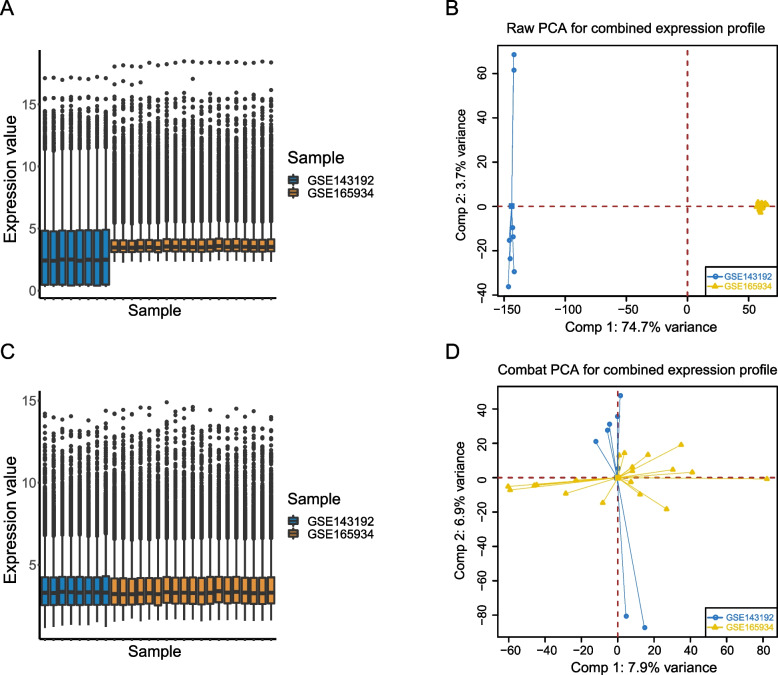
Quality assessment of GSE143192 and GSE165934 before and after the batch effect correction. **A** Box plot before batch effect correction. **B** Principal Components Analysis (PCA) plot before batch effect correction. **C** Box plot after batch effect correction. **D** PCA plot after batch effect correction

After batch effect correction and integration of the GSE143192 and GSE165934 datasets, we obtained 12,125 and 3,376 common lncRNAs and mRNAs, respectively. We evaluated data quality before and after batch effect correction and generated box plots (Figs. 2A and C) and PCA plots (Figs. 2B and D) of the lncRNA expression profile before and after the batch effect correction. The results showed that the expression of genes in the two datasets before correction was unevenly distributed with significant principal component distinction (Figs. 2A and B). In contrast, the expression of genes in the two datasets after correction was evenly distributed with no significant principal component distinction (Figs. 2C and D), indicating that the batch effect was effectively reduced, which could improve subsequent data analysis.

### Screening for differentially expressed lncRNAs and construction of ceRNA networks

**Fig. 3:**
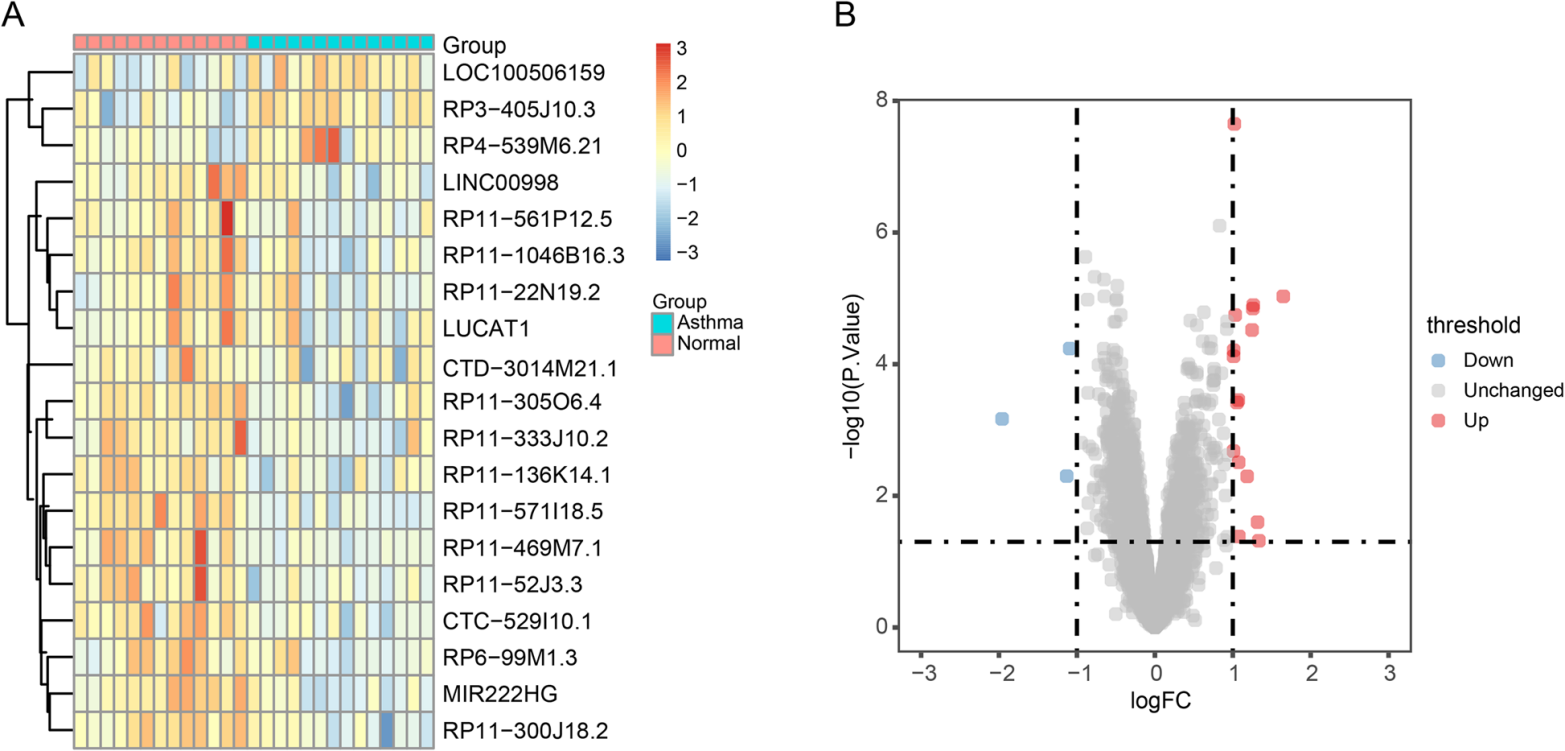
Differential lncRNA analysis of the dataset after batch effect correction. **A** Heatmap showed the relative level of 19 upregulated and downregulated differentially expressed lncRNAs (DElncRNAs) in in the dataset after batch effect correction,with up-regulated lncRNAs (red) and down-regulated lncRNAs (blue). **B** Volcano plot of DElncRNAs in the dataset after batch effect correction, with high expression (red) and low expression (blue)

**Fig. 4:**
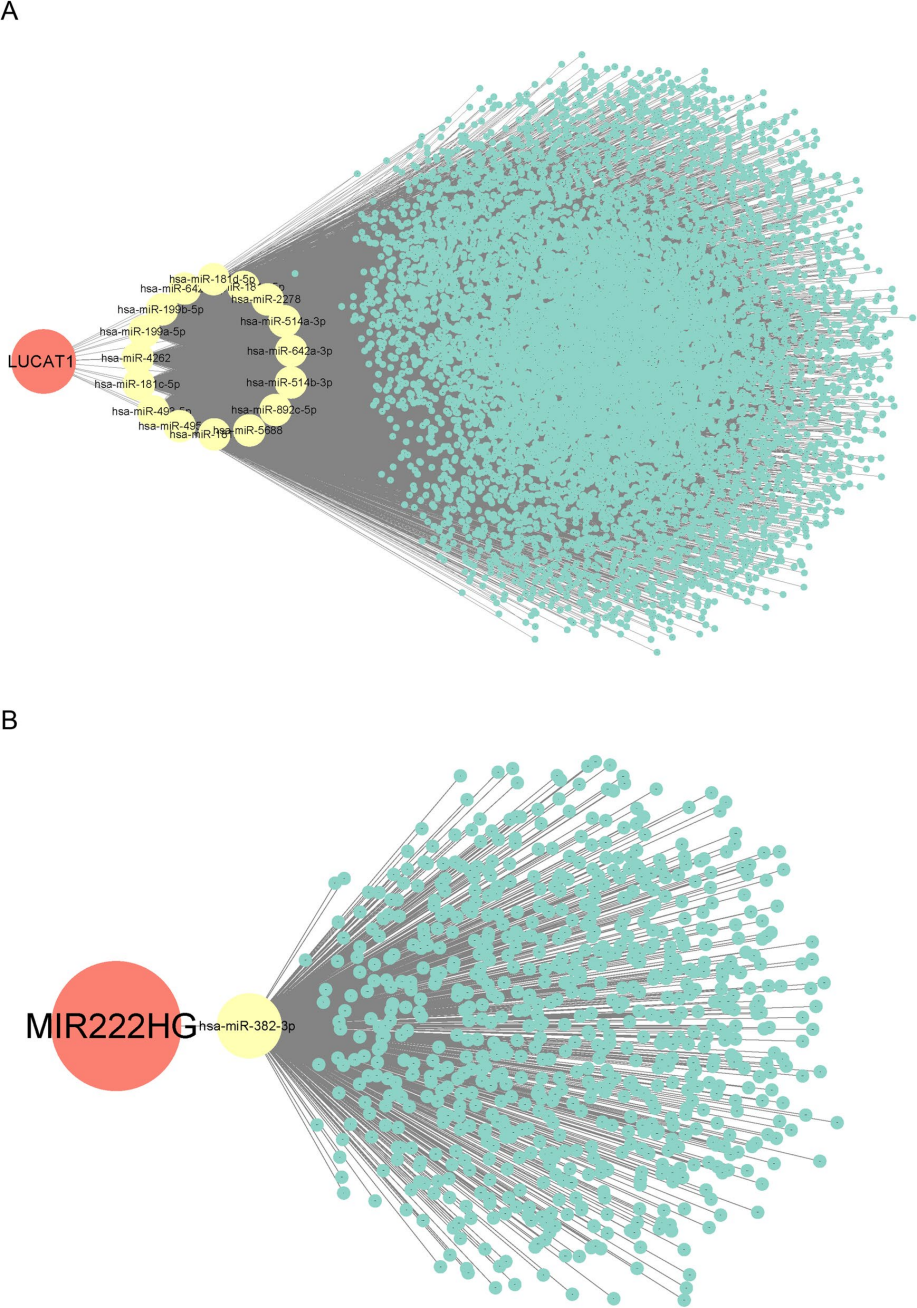
Competing endogenous RNA (CeRNA) networks for LUCAT1 and MIR222HG, with red dots for lncRNA, yellow dots for miRNA, and green dots for mRNA. **A** CeRNA network for LUCAT1. **B** CeRNA network for MIR222HG

We performed variance analysis on the batch effect-corrected lncRNA expression profile data and obtained 19 DElncRNAs. The heat map (Fig. 3A) and volcano plot (Fig. 3B) allowed us to visualize the differentially expressed lncRNAs between patients with asthma and healthy individuals. Next, the results of the DElncRNA-miRNA-mRNA interaction ceRNA regulatory network showed that only two lncRNAs, namely, LUCAT1 and MIR222HG, had corresponding target miRNAs in the StarBase database. Therefore, we constructed lncRNA-miRNA-mRNA axes for LUCAT1 and MIR222HG, respectively. LUCAT1 had 16 target miRNAs, and 9136 mRNAs were targeted by these 16 miRNAs (Fig. 4A and Table S2); MIR222HG had only one target miRNA (has-miR-382-3p), and 756 mRNAs were targeted by this miRNA (Fig. 4B and Table S3).

### Ferroptosis-related DEG enrichment analysis

**Fig. 5:**
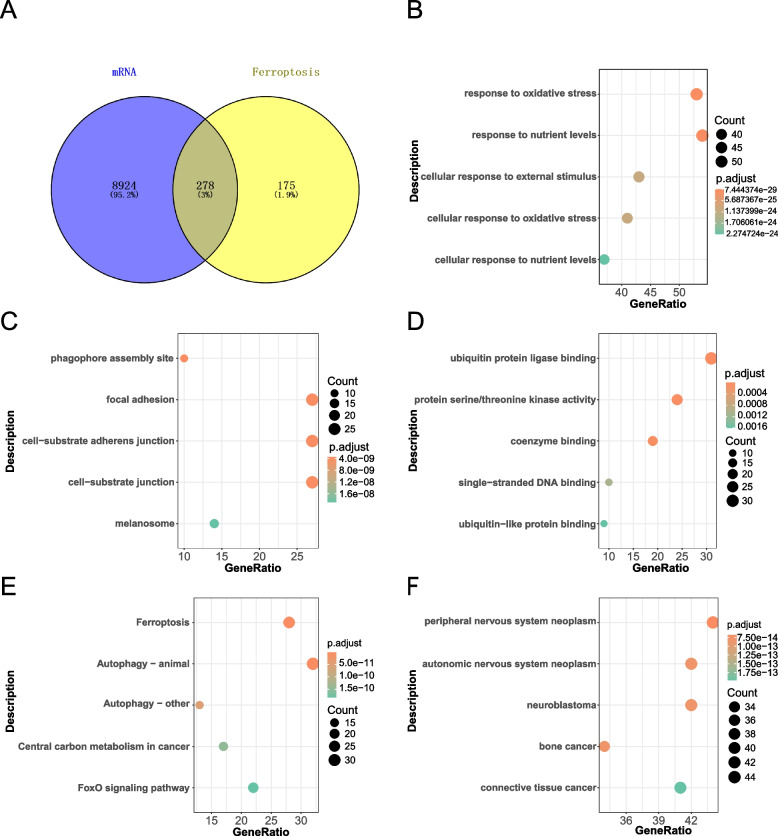
GO, KEGG and DO enrichment analysis results of 278 differential ferroptosis–related genes. **A** Venn diagram showed 278 overlapping genes of mRNAs targeted by lncRNAs and ferroptosis–related genes. **B** Bubble plot of GO-BP enrichment results in differential ferroptosis–related genes. **C** Bubble plot of GO-CC enrichment results in differential ferroptosis–related genes. **D** Bubble plot of GO-MF enrichment results in differential ferroptosis–related genes. **E** Bubble plot of KEGG enrichment results in differential ferroptosis–related genes. **F** Bubble plot of DO enrichment results in differential ferroptosis–related genes

The target mRNAs of LUCAT1 and MIR222HG obtained in the previous procedure (a total of 9202 genes) were compared with ferroptosis-related genes (Fig. 5A) to obtain 278 differential ferroptosis-related genes. GO enrichment analysis revealed that these differential ferroptosis-related genes were significantly enriched during biological processes, including response to oxidative stress, response to nutrient levels, cellular response to external stimulus, cellular components (such as phagophore assembly sites, focal adhesion, cell-substrate junction, and melanosome), and molecular functions (such as ubiquitin protein ligase binding, protein serine/threonine kinase activity, coenzyme binding, and single-stranded DNA binding). KEGG pathway enrichment analysis showed that these differential genes were significantly enriched in signaling pathways, including ferroptosis, autophagy, central carbon metabolism in cancer, FoxO signaling pathway, and mitophagy. Moreover, DO enrichment analysis revealed that these differential genes were involved in peripheral nervous system neoplasm, autonomic nervous system neoplasm, neuroblastoma, and bone cancer. We selected the top five differentials ferroptosis-related genes from the enrichment analysis results to generate bubble plots for GO-BP, GO-CC, and GO-MF (Figs. 5B-D) and KEGG and DO analyses (Figs. 5E and F). The detailed GO enrichment analysis results are shown in Table 2, and the KEGG and DO enrichment analysis results are shown in Table 3.

**Table 2:** GO enrichment analysis of ferroptosis-related differentially expressed genes (DEGs)

**ID**	**Description**	**Count in gene set**	***p*-value**
**GO-BP**			
GO:0006979	response to oxidative stress	53	1.66E-32
GO:0031667	response to nutrient levels	54	2.78E-31
GO:0071496	cellular response to external stimulus	43	9.36E-28
GO:0034599	cellular response to oxidative stress	41	1.16E-27
GO:0031669	cellular response to nutrient levels	37	3.05E-27
GO:2001233	regulation of apoptotic signaling pathway	41	1.25E-22
GO:0010038	response to metal ion	38	1.58E-21
GO:0006914	autophagy	41	2.19E-19
GO:0061919	process utilizing autophagic mechanism	41	2.19E-19
GO:0035690	cellular response to drug	35	1.54E-18
**GO-CC**			
GO:0000407	phagophore assembly site	10	1.21E-11
GO:0005925	focal adhesion	27	2.16E-11
GO:0005924	cell-substrate adherens junction	27	2.55E-11
GO:0030055	cell-substrate junction	27	3.19E-11
GO:0042470	melanosome	14	2.67E-10
GO:0048770	pigment granule	14	2.67E-10
GO:0005776	autophagosome	12	6.78E-09
GO:1902911	protein kinase complex	12	4.18E-08
GO:0034045	phagophore assembly site membrane	6	5.07E-08
GO:0031968	organelle outer membrane	15	1.64E-07
**GO-MF**			
GO:0031625	ubiquitin protein ligase binding	31	2.27E-17
GO:0004674	protein serine/threonine kinase activity	24	1.03E-07
GO:0050662	coenzyme binding	19	1.56E-07
GO:0003697	single-stranded DNA binding	10	1.06E-05
GO:0032182	ubiquitin-like protein binding	9	1.85E-05
GO:0008198	ferrous iron binding	5	2.92E-05
GO:0051219	phosphoprotein binding	8	4.42E-05
GO:0003996	acyl-CoA ligase activity	4	8.96E-05
GO:0016874	ligase activity	10	9.11E-05
GO:0015175	neutral amino acid transmembrane transporter activity	5	0.000107

**Table 3:** KEGG and DO enrichment analyses of ferroptosis-related DEGs

**ID**	**Description**	**Count in gene set**	***p*-value**
**KEGG**			
hsa04216	Ferroptosis	28	1.91E-35
hsa04140	Autophagy—animal	32	1.79E-21
hsa04136	Autophagy—other	13	5.40E-13
hsa05230	Central carbon metabolism in cancer	17	2.13E-12
hsa04068	FoxO signaling pathway	22	3.34E-12
hsa04137	Mitophagy—animal	16	4.18E-11
hsa05167	Kaposi sarcoma-associated herpesvirus infection	25	4.45E-11
hsa04066	HIF-1 signaling pathway	19	5.49E-11
hsa04621	NOD-like receptor signaling pathway	24	8.65E-11
hsa05161	Hepatitis B	22	2.51E-10
**DO**			
DOID:1192	peripheral nervous system neoplasm	44	8.98E-17
DOID:2621	autonomic nervous system neoplasm	42	3.81E-16
DOID:769	neuroblastoma	42	3.81E-16
DOID:184	bone cancer	34	4.36E-16
DOID:201	connective tissue cancer	41	1.33E-15
DOID:0050736	autosomal dominant disease	43	1.65E-15
DOID:3347	osteosarcoma	31	4.22E-15
DOID:4450	renal cell carcinoma	38	1.57E-14
DOID:3996	urinary system cancer	46	2.33E-14
DOID:263	kidney cancer	42	8.72E-14

### GSEA and GSVA analyses of the mRNA expression profile

**Fig. 6:**
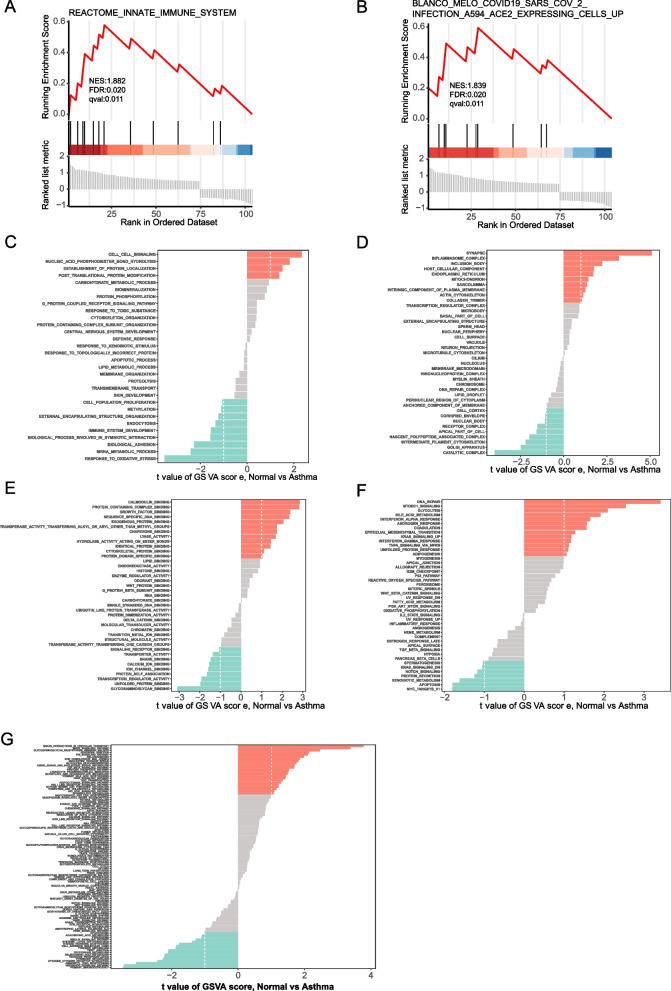
Plots of GSEA and GSVA results. **A** GSEA-enriched gene set REACTOME_INNATE_IMMUNE_SYSTEM. **B** GSEA-enriched gene set BLANCO_MELO_COVID19_SARS_COV_2_INFECTION_A594_ACE2_EXPRESSING_CELLS_UP. **C** Histogram of upregulated and downregulated gene sets in GSVA in the GO-BP reference set. **D** Histogram of GSVA enrichment results in the GO-CC reference set. **E** Histogram of GSVA enrichment results in the GO-MF reference set. **F** Histogram of GSVA enrichment results in the Hallmarker reference set. **G** Histogram of GSVA enrichment results in the KEGG reference set

The GSEA result against the mRNA expression profile of asthma showed a significant correlation with gene sets, including “REACTOME_INNATE_IMMUNE_SYSTEM” and “BLANCO_MELO_COVID19_SARS_COV2_INFECTION_A594_ACE2_EXPRESSING_CELLS_UP” data sets (Figs. 6A and B). Meanwhile, the GSVA results primarily included the results of KEGG, GO (GO-BP, GO-CC, and GO-MF), and Hallmarker enrichment analyses. The top five highest GSVA values in GO-BP enrichment analysis results were associated with response to oxidative stress, mRNA metabolic process, cell–cell signaling, bio-adhesion, and nucleic acid phosphodiester bond hydrolysis. The GO-CC results showed that the relevant differential gene products primarily functioned in cellular components, including synapse, catalytic complex, inflammasome complex, Golgi apparatus, and inclusion body. The main results of GO-MF analysis include glycosaminoglycan binding, calmodulin binding, protein-containing complex binding, growth factor binding, and sequence-specific DNA binding. The results of the Hallmarker enrichment analysis included DNA repair, MTORC1 signaling, glycolysis, bile acid metabolism, and MYC-targeted v1. The results of the KEGG enrichment analysis primarily included SNARE interactions in vesicular transport, primary immunodeficiency, the Hedgehog signaling pathway, MAPK signaling pathway, and the aminoacyl tRNA biosynthesis pathway. We plotted histograms of upregulated and downregulated differential genes in gene sets based on the GSVA results (Figs. 6C-G). The GSEA results are shown in Table 4, and the GSVA results are shown in Table 5.

**Table 4:** GSEA results of the mRNA expression profile

GSEA			
**ID**	**NES**	***p*-adjust**	***q*-value**
REACTOME_INNATE_IMMUNE_SYSTEM	1.882402	0.02038	0.010727
BLANCO_MELO_COVID19_SARS_COV_2_INFECTION_A594_ACE2_EXPRESSING_CELLS_UP	1.839356	0.02038	0.010727

**Table 5:** GSVA results of the mRNA expression profile

Gene Set	ID	Score
KEGG	SNARE_INTERACTIONS_IN_VESICULAR_TRANSPORT	3.779257
KEGG	PRIMARY_IMMUNODEFICIENCY	-3.4665
KEGG	HEDGEHOG_SIGNALING_PATHWAY	-3.4328
KEGG	MAPK_SIGNALING_PATHWAY	3.381272
KEGG	AMINOACYL_TRNA_BIOSYNTHESIS	-3.07139
GO-BP	RESPONSE_TO_OXIDATIVE_STRESS	-3.52922
GO-BP	MRNA_METABOLIC_PROCESS	-2.41036
GO-BP	CELL_CELL_SIGNALING	2.343272
GO-BP	BIOLOGICAL_ADHESION	-2.26262
GO-BP	NUCLEIC_ACID_PHOSPHODIESTER_BOND_HYDROLYSIS	1.82118
GO-CC	SYNAPSE	5.093992
GO-CC	CATALYTIC_COMPLEX	-3.99279
GO-CC	INFLAMMASOME_COMPLEX	3.192443
GO-CC	GOLGI_APPARATUS	-2.481
GO-CC	INCLUSION_BODY	2.224472
GO-MF	GLYCOSAMINOGLYCAN_BINDING	-3.08434
GO-MF	CALMODULIN_BINDING	2.854325
GO-MF	PROTEIN_CONTAINING_COMPLEX_BINDING	2.83028
GO-MF	GROWTH_FACTOR_BINDING	2.393979
GO-MF	SEQUENCE_SPECIFIC_DNA_BINDING	2.341345
Hallmarker	DNA_REPAIR	3.412268
Hallmarker	MTORC1_SIGNALING	2.539293
Hallmarker	GLYCOLYSIS	2.092539
Hallmarker	BILE_ACID_METABOLISM	1.911157
Hallmarker	MYC_TARGETS_V1	-1.80191

### Biomarker screening by LASSO regression

**Fig. 7:**
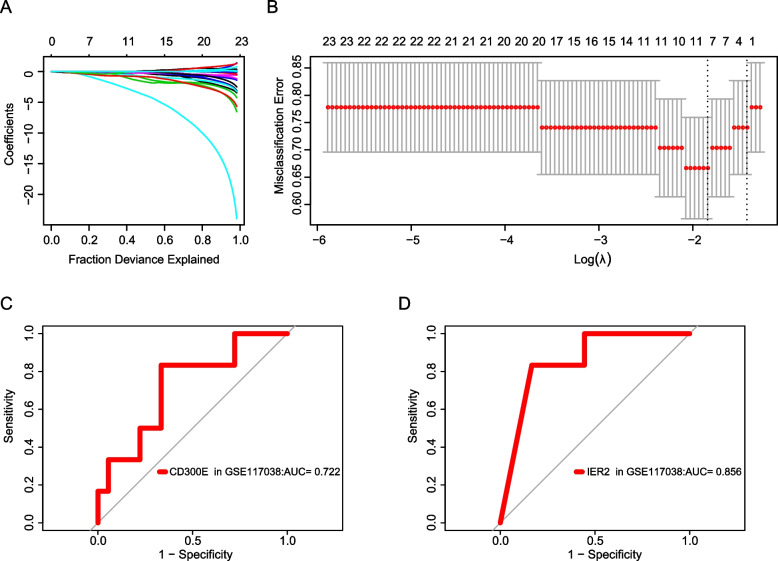
Biomarkers screened by the Least Absolute Shrinkage and Selection Operator (LASSO) regression algorithm. **A** The LASSO regression algorithm in training set. **B** The optimal value for penalization coefficient λ in training set. **C** ROC curve of CD300E in validation set. **D** ROC curve of IER2 in validation set

Based on the constructed LASSO-penalized regression model (Fig. 7A), the number of variables was determined by finding λ with the lowest classification error (λ = 0.1583217) (Fig. 7B). Consequently, we screened seven potential marker genes (BCL10, CD300E, IER2, MMP13, OAF, TBC1D3, and TMEM151A). The ROC curve results plotted based on these seven candidate genes showed that only CD300E and IER2 featured good diagnostic effects in the validation set GSE117038, with AUC values of 0.722 and 0.856, respectively (Figs. 7C and D). These results indicate that CD300E and IER2 may serve as novel asthma biomarkers.

### Immuno-infiltration analysis by CIBERSORT

**Fig. 8:**
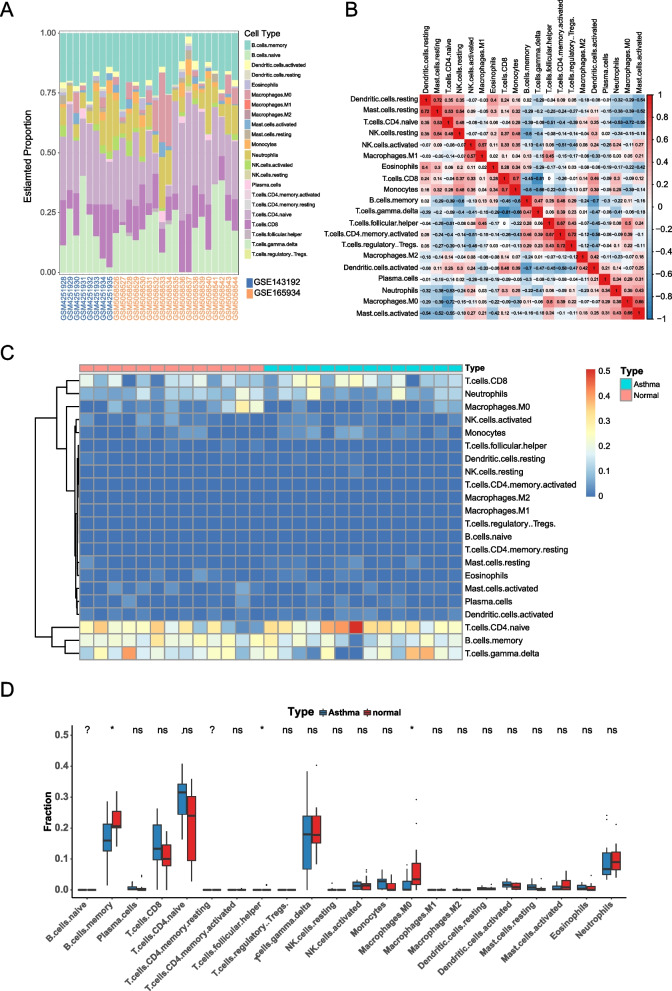
Immune cell infiltration assessment and visualization results. **A** Bar chart of immune cell infiltration distribution across different samples in GSE143192 and GSE165934. **B** Correlation heat map of 20 types of infiltrating immune cells, with red representing positive correlation and blue representing negative correlation; the darker the color, the stronger the correlation. **C** Heat map of immune cells differentially expressed between asthma and normal cohorts, with red bands indicating high expression and blue representing low expression. **D** Box plot of the content of different immune cells between asthma and normal cohorts, with blue representing asthma samples and red representing normal samples,*p-value < 0.05

We analyzed the changes in the proportion of various infiltrating immune cells in the GSE143192 and GSE165934 datasets using CIBERSORT (Fig. 8A). The results showed differences in immune cell composition among different groups. The correlation heat map of 20 types of immune cells (the algorithm identified a total of 22 types of immune cells, but the immune infiltration results of resting CD4 + memory T cells and naive B cells were null) showed (Fig. 8B) that naive CD4 + T cells were significantly and positively correlated with resting dendritic cells and resting mast cells. Meanwhile, such immune cells were significantly but negatively correlated with T follicular helper cells and activated CD4 + memory T cells. The heat map results of the variance analysis exhibited (Fig. 8C) that some immune cells differed in number between patients with asthma and normal groups. Meanwhile, the box plot results indicated (Fig. 8D) that the number of memory B cells, CD8 + T cells, naive CD4 + T cells, and M0 macrophages significantly differed between patients with asthma and normal groups.

### Correlation analysis of key genes and infiltrating immune cells

**Fig. 9:**
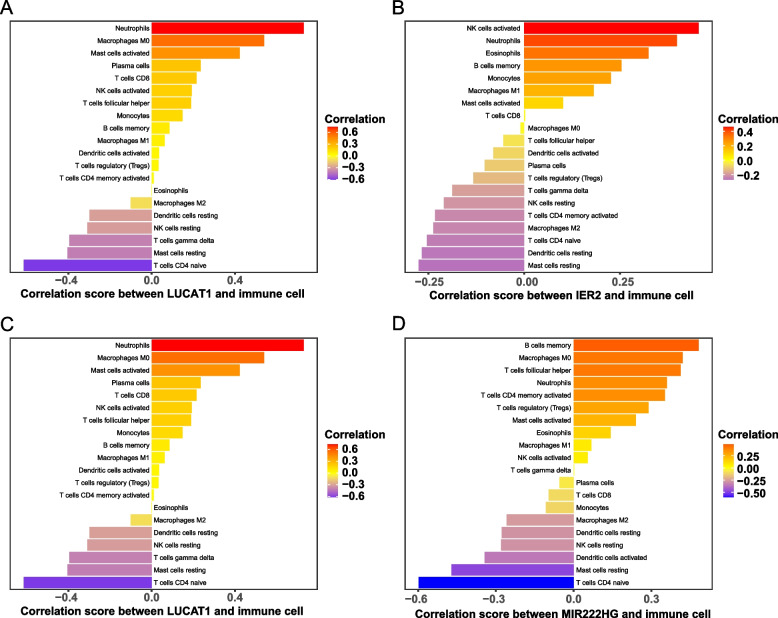
The correlation analysis results of key genes with immune cells. **A** Lollipop plot of the correlation between LUCAT1 in the mRNA expression profile and immune cells. **B** Lollipop plot of the correlation between MIR222HG in the mRNA expression profile and immune cells. **C** Lollipop plot of the correlation between CD300E in the mRNA expression profile and immune cells. **D** Lollipop plot of the correlation between IER2 in the mRNA expression profile and immune cells

Finally, we investigated the association between the immune cell ratio and the expression of LUCAT1, MIR222HG, CD300E, and IER2 in patients with asthma to determine the biomarkers correlated with the immune cell ratio. The lollipop plots showed the correlation of LUCAT1, MIE222HG, CD300E, and IER2 with immune cells (Fig. 9). The results of immune cells with significant correlation (p-value < 0.05) showed that LUCAT1 expression was positively correlated with the ratios of M0 macrophages, activated mast cells, and neutrophils and negatively correlated with the ratios of resting mast cells, naive CD4 + T cells, and γ-δ T cells; MIR222HG expression was positively correlated with the ratios of memory B cells, M0 macrophages, and T follicular helper cells and negatively correlated with the ratios of resting mast cells and naive CD4 + T cells; CD300E expression was positively correlated with the ratios of M1 macrophages and activated NK cells; IER2 expression was positively correlated with the ratios of neutrophils and activated NK cells.

## Discussion

Asthma is a global problem affecting people of all ages. However, the current clinical diagnosis of asthma is usually based on clinical symptoms and variable and reversible airflow limitations, with no clear diagnostic gold standard. Although phenotypic analysis based on airway inflammatory biomarkers has elucidated the pathophysiological mechanism of asthma [39], the actual control of asthma remains suboptimal, with treatments mostly based on anti-inflammatory/bronchodilator regimens. Meanwhile, increasing studies have shown that lncRNAs serve as ceRNAs to regulate the onset and progression of asthma [40–42]. Therefore, we could make implications for achieving an early diagnosis and treatment of asthma by identifying novel biomarkers and targets of asthma and exploring the relationship between asthma and ferroptosis, as well as the diversity and complexity of the immune microenvironment.

In this study, we initially screened the differentially expressed lncRNAs using the StarBase database to obtain lncRNAs with targeted miRNAs (LUCAT1 and MIR222HG) and then constructed ceRNA networks by predicting targeting mRNAs from these miRNAs. LncRNAs are non-coding RNAs with a length of > 200 nucleotides, which account for nearly 70% of the human transcriptome. They are highly important in regulating almost all biological processes, and they serve as important regulators of histophysiology and disease processes [43]. LncRNAs also play a role in regulating the progression of asthma.

LUCAT1, which is also known as smoke and cancer-associated lncRNA1 [44], is highly expressed in various malignancies. It regulates the proliferation, migration, and invasion of malignant tumors through diverse mechanisms [45, 46]. Furthermore, studies have demonstrated that elevated LUCAT1 levels suppressed the expression of inflammatory genes and interferon-stimulated genes [47], whereas the overexpression of LUCAT1 in human lung cell lines cultured in high-glucose conditions resulted in reduced iNOS and NO levels [48]. In particular, LUCAT1 could regulate anti-inflammatory processes through different mechanisms. In the last two years, several studies that used bioinformatic analysis to screen key genes have shown that LUCAT1 is a differentially expressed lncRNA associated with ferroptosis [49, 50]. However, MIR222HG, which belongs to the lncRNA subclass of miRNA host genes and is a miR222/221 cluster host gene, remains poorly understood by researchers [51]. MIR222HG expression promotes the development of castration-resistant prostate cancer [52], whereas it was screened in an investigation of whether immune-related lncRNAs could be used as diagnostic markers for glioblastoma. In addition, the MIR222HG-ILF3 RNP complex regulates the RNA stability of DNM3OS, a cell cycle regulator [51]. However, neither LUCAT1 nor MIR222HG has been studied in the context of asthma remains unknown.

In recent years, there has been growing interest regarding the importance of ferroptosis in asthma, and lncRNAs are emerging as key mediators in ferroptosis studies [53]. For example, LINC00618 inhibits ferroptosis by attenuating the expression of lymphatic-like specific helicase and SLC7A11 [54]. LINC00336 also serves as a ceRNA to inhibit ferroptosis by binding to MIR6852 to regulate cystathionine-beta-synthase expression [55]. In this work, we obtained ferroptosis–related differential genes through ceRNA networks constructed by differentially expressed lncRNAs. The preliminary exploration of their enrichment analysis might provide insights into further research on the ferroptosis–related lncRNA-miRNA-mRNA regulatory axis. GO enrichment analysis revealed that these differential ferroptosis-related genes are closely associated with oxidative stress, which is consistent with the definition of ferroptosis (iron and oxidative stress-dependent programmed cell death), while the biochemical features of ferroptosis primarily include increased cellular unstable iron, massive ROS production, decreased GPX4, and lipid metabolite accumulation [56]. Meanwhile, recent studies have found that oxidative/antioxidative imbalance and increased oxidative stress products contribute to airway inflammation, mucus hypersecretion, and airway remodeling, thereby playing a role in asthma progression [57, 58]. Therefore, the role of oxidative stress in asthma has also received increasing attention from researchers. In this study, the KEGG analysis revealed that apart from being highly involved in the ferroptosis pathway, these differential ferroptosis–related genes were also significantly enriched in signaling pathways, including autophagy, FoxO signaling, and mitophagy. In addition, ferroptosis, autophagy, and mitophagy are closely related to each other [59, 60], affecting asthma progression under the regulation of different mechanisms. For example, the PEBP1/15-LO1 complex, which mediates the onset of ferroptosis, is elevated in patients with asthma, which induces airway redox imbalance, thereby causing T2 inflammation and asthma exacerbation [14]; MIR-335-5P targets and regulates ATG5, which reduces the onset of autophagy in the airways of patients with asthma, attenuating an inflammatory response [61]; estrogen receptor 2 transcriptionally suppresses the expression of miRNA-423, which increases the expression level of PINK1 in asthma and mitophagy mediated via PINK1, leading to the worsening of asthma [62]. A variety of programmed cell death pathways may interact with one another during asthma pathogenesis; however, this needs further exploration. Moreover, ferroptosis-related genes were enriched in the FoxO signaling pathway in a biomedical study on amyotrophic lateral sclerosis [63]. Christina et al*.* showed in their study using a Drosophila model that targeting the JNK/FoxO signaling pathway could regulate airway remodeling in chronic inflammatory lung diseases [64]. Furthermore, elevated FoxO1 expression in airway macrophages among patients with mild asthma induces macrophage polarization in the lungs, which is involved in airway inflammation and airway remodeling in asthma [65]. LUCAT1 and MIR222HG may modulate ferroptosis in asthma by various mechanisms, thereby influencing asthma progression. Analysis based on GSEA found that the innate immune system genes were significantly affected. Different form the GO and KEGG enrichment analyses of ferroptosis-related DEGs, GSVA was able to use whole-genome information and further distinguish the differences in biological behaviors between patients and healthy subjects.

In the present study, we screened seven key genes using the LASSO algorithm and identified differentially expressed genes, namely, CD300E and IER2, with AUC values greater than 0.7 as diagnostic markers for asthma using ROC curve analysis. CD300E, originally known as the immune receptor expressed by myeloid cells (IREM)-2, is a glycosylated surface receptor primarily expressed in human monocytes and myeloid DCs, which serves as an activating receptor that regulates inflammatory and immune responses [66, 67]. Immediate early response 2 (IER2) is a potential DNA-binding protein that serves as a transcription factor or transcriptional co-activator in regulating cellular biological processes [68]. Although CD300E and IER2 have not been reported in the context of asthma, CD300E serves as a biomarker for M2c macrophages [69]. Additionally, the level of M2c macrophages can be used as a marker for asthma to detect the severity of the disease and guide treatment strategies [70]. Therefore, subsequent studies must be conducted to validate the conjecture of CD300E as a diagnostic marker for asthma. Although we initially revealed several potential biomarkers using machine learning in this study that have a high diagnostic value for asthma, further examination of CD300E and IER2 expression levels in hospital-constructed asthma cohorts is necessary to verify the diagnostic power of these marker genes before they can be clinically used.

Finally, we applied the integrated genetic dataset to assess the differences in immune cell composition patterns between patients with asthma and healthy individuals by CIBERSORT. Among the 22 types of immune cells, CD4 + T cells, B cells, and mast cells showed significant differences between the two groups, which is consistent with previous reports on immune cell migration and infiltration in asthma [71–73]. In addition, we found a correlation between the infiltration of certain immune cells and the expression of key genes. We hypothesized that these genes may boost the abundance of immune cells through certain mechanisms, thereby influencing the progression of asthma. However, this correlation remains unclear, which requires validation by in vitro and in vivo experiments, which might be the endpoint of further studies. Meanwhile, we cannot exclude the possibility that there is no causal relationship between immune cell infiltration and the expression of these marker genes and that airway inflammation is responsible for the changes in immune cell ratios and marker gene expression during asthma progression.

This study also has some limitations: First, the small dataset collected from the GEO database and the relatively small sample size used for analysis and validation might lead to bias in the analysis of key genes and CIBERSORT. Next, the immune infiltration analysis based on PBMC samples may have some limitations due to potential interfering factors. In addition, our data analysis was based on open-source data sets, and the key genes screened by the analysis were not adequately supported by the literature, indicating that the credibility of the results should be verified in further experiments.

## Conclusion

In this study, we screened key asthma-related genes (LUCAT1, MIR222HG, CD300E, and IER2) and immune cell infiltration profiles through bioinformatic analysis, which could provide insights into the pathogenesis of asthma and new perspectives and approaches for asthma diagnosis and treatment.

## Supplementary Information

**Additional file 1: Table S1.** List of ferroptosis-related genes.

**Additional file 2: Table S2.** List of miRNAs and mRNAs targeted by LUCAT1.

**Additional file 3: Table S3.** List of miRNAs and mRNAs targeted by MIR222HG.

## Data Availability

Com Publicly available datasets were analyzed in this study, which can be found in the Gene Expression Omnibus (https://www.ncbi.nlm.nih.gov), MSigDB database (https://www.gsea-msigdb.org/gsea/msigdb), GeneCards database (http://www.genecards.org), FerrDb database (http://www.zhounan.org/ferrdb/), and StarBase database (http://StarBase.sysu.edu.cn/).

## Abbreviations

ceRNA: Competing endogenous RNA
GEO: Gene Expression Omnibus
DO: Disease Ontology
GO: Gene Ontology
KEGG: Kyoto Encyclopedia of Genes and Genomes
GSEA: Gene Set Enrichment Analysis
GSVA: Gene Set Variation Analysis
LASSO: Least Absolute Shrinkage and Selection Operator
ROC: Receiver operating characteristic
AUC: Area under the curve
GAN1: Global Asthma Network 1
lncRNA: Long non-coding RNA
circRNA: Circular RNA
MRE: MiRNA response elements
LOX: Lipoxygenase
COX: Cyclooxygenase
PEBP1/15-LO: Phosphatidylethanolamine-binding protein 1/15 LOX complex
HAEC: Human airway epithelial cell
GPX4: Glutathione peroxidase 4
SLC7A11: Solute carrier family 7 member 11
DFO: Deferoxamine
Fer-1: Ferrostatin-1
DC: Dendritic cell
LUCAT1: Lung cancer–associated transcript 1
IREM: Immune receptor expressed by myeloid cells
IER2: Immediate early response 2
